# Chronic Alcohol Consumption Induces Irreversible and Heterogeneous Pancreatic Steatosis in Men: An MRI-Based Cross-Sectional Study

**DOI:** 10.3390/jcm15072513

**Published:** 2026-03-25

**Authors:** Yuting Zhao, Wenjuan Yang, Chengwei Tang, Jing Li

**Affiliations:** Department of Gastroenterology and Hepatology, West China Hospital, Sichuan University, Chengdu 610041, China; 13308919040@163.com (Y.Z.); yangwenjuan19860@163.com (W.Y.); shcqcdmed@163.com (C.T.)

**Keywords:** alcohol, pancreatic steatosis, MRI, abstinence, fat distribution, male population, fat metabolism, irreversible damage

## Abstract

**Background:** Chronic alcohol consumption is a recognized cause of pancreatic steatosis, though its imaging characteristics in individuals remain undefined. This study investigated the effect of chronic alcohol intake on the pancreatic fat content and distribution in a male population and assessed the impact of abstinence. **Methods:** In this cross-sectional study, 140 male alcohol drinkers (who consumed >20 g ethanol/day for >5 years) and 142 male non-drinkers were recruited. The pancreatic fat fraction (FF) was quantified using T1- and T2*-corrected double-echo chemical shift magnetic resonance imaging (CSI). Participants were stratified by age (20–50 years, 50–70 years). Drinkers were further categorized as current drinkers or short-term (<1 year) or long-term (1–2 years) abstainers. **Results:** The pancreatic FF was significantly higher in alcohol drinkers compared with age-matched controls in the 20–50-year-old (6.07 ± 1.59% vs. 2.94 ± 0.62%, *p* < 0.05) and 50–70-year-old (9.14 ± 2.22% vs. 5.98 ± 1.00%, *p* < 0.05) groups. In drinkers aged 40–70 years, the pancreatic fat distribution was uneven. No significant difference in the FF was observed across the three drinking status groups (*p* > 0.05). **Conclusions:** Chronic alcohol consumption could cause fat deposition in the pancreas. An uneven distribution of pancreatic fat began in the fourth decade in this male population. An alcoholic fatty pancreas was not reversed within a follow-up period of up to two years.

## 1. Introduction

Fatty liver disease has garnered substantial clinical and scientific attention, yet fatty pancreas—despite being described in the autopsy literature as early as 1933—remains conspicuously understudied as an independent pathological condition [1]. Rather than being viewed as a primary pathological condition, pancreatic steatosis has often been relegated to the status of a secondary complication of disorders such as type 2 diabetes, obesity, pancreatitis, and pancreatic cancer [2,3,4,5,6]. In fact, fatty pancreas is a clinical pathological syndrome characterized by fat deposition in pancreatic tissue, with many causes such as heredity, environment, and metabolism. It is not a benign condition; rather, it leads to a series of adverse consequences. A growing body of literature has shown that excessive fat deposition in the pancreas not only causes exocrine and endocrine pancreatic function injury but also increases susceptibility to acute pancreatitis and pancreatic cancer [7,8,9,10,11,12,13,14,15,16,17]. Pancreatic steatosis has been identified as an independent risk factor for chronic pancreatitis, type 2 diabetes, and pancreatic ductal adenocarcinoma [7,8,9,16], making it a key clinical concern that requires further research to guide prevention and management strategies.

Among the various etiologies, chronic alcohol consumption is considered one of the most common causes of fatty pancreas. The molecular mechanism of alcohol-related pancreatic steatosis involves dysregulation of lipid metabolism. Chronic ethanol exposure can activate SREBP-1c to promote fatty acid synthesis and inhibit ATGL/HSL to reduce lipolysis, thereby exacerbating lipid accumulation [18,19,20]. These mechanisms are similar to alcoholic fatty liver. However, the pancreas lacks efficient lipid delivery systems, such as VLDL, resulting in more persistent fat deposition [21]. Unfortunately, the existing literature on alcoholic fatty pancreas has been largely limited to animals and autopsies [1,18]. Our previous animal studies demonstrated that chronic alcohol intake was closely related to a pronounced accumulation of lipid droplets within pancreatic acinar cells and significantly increased triglyceride content of the pancreas in rats [18]. In humans, the only reported long-term alcohol-induced pancreatic steatosis was found during an autopsy [1]. However, autopsy findings may not accurately reflect the actual pancreatic changes that result from alcohol intake in living individuals, as the pancreas is especially vulnerable to ischemia and hypoxia. To our knowledge, alcoholic pancreatic steatosis has not been investigated in living humans using modern imaging techniques. Furthermore, while physiological pancreatic fat deposition (e.g., aging-related fat accumulation) usually presents as a homogeneous distribution in healthy populations [22,23], focal or heterogeneous fat accumulation is considered pathological and has been reported in several pancreatic diseases, including chronic pancreatitis, cystic fibrosis, and pancreatic cancer [24,25,26]. The distribution characteristics of alcohol-induced pancreatic fat deposition in humans remain unknown. Additionally, although abstinence is recommended as an effective treatment for alcoholic fatty liver, our previous animal study suggested that alcoholic pancreatic steatosis did not attenuate after alcohol withdrawal [12]. The effect of abstinence on alcohol-induced pancreatic fat deposition in humans still needs to be explored.

To address these unanswered questions in living humans, a reliable non-invasive quantification method is essential. In recent years, T1- and T2*-corrected double-echo chemical shift magnetic resonance imaging (CSI) has gained widespread acceptance in the clinic for quantification of pancreatic fat content with its characteristics of rapidity and high accuracy [22,27]. Our group has successfully quantified the pancreatic fat fraction in healthy populations in previous studies [22,23,27]. Although magnetic resonance spectroscopy, multi-echo technique, and iterative decomposition of water and fat with echo asymmetry and least squares estimation quantification sequence are also available effective techniques for fat quantification, they are not convenient for a busy clinical practice nor suitable for pancreatic fat quantification because the pancreatic region of interest (ROI) is too small to ensure a high accuracy due to the interference of respiratory and homogeneity of the magnetic field [22,23,27]. Therefore, this study aimed to investigate whether chronic alcohol intake causes heterogeneous fat deposition in the male pancreas and assess the potential for diversity following alcohol abstinence.

## 2. Methods

### 2.1. Volunteers

For the present study, a total of 142 male non-drinkers and 140 male alcoholics were recruited from the outpatient department of West China Hospital of Sichuan University from January 2018 to June 2020. The inclusion and exclusion criteria are detailed in Supplementary Table S1. Enrolled non-drinkers had no history of long-term or excess alcohol consumption (>20 g ethanol/day for at least one year). Alcoholics were defined as people who drank for more than 5 years with consumption of at least 20 g of ethanol in one day and did not stop drinking for more than 2 years. The alcohol consumption was self-reported. The body mass index (BMI) was limited to 18~25 kg/m^2^ because obesity is a risk factor for fatty pancreas. All participants were divided into four age groups: 20~30 years, 30~40 years, 40~50 years, and 50~70 years. Non-drinkers and alcoholics served as the control and alcohol groups, respectively. In addition, alcoholics were divided into current drinkers, short-term abstainers (<1 year), and long-term abstainers (1~2 years) depending on whether the volunteer had already stopped drinking and the length of abstinence. The study was approved by the Ethics Committee of West China Hospital of Sichuan University and was registered on the Chinese Clinical Trial Register (registration number: ChiCTR-CCH-00000147).

### 2.2. Phantom Construction of Fat–Water Mixture

A fat emulsion at a concentration of 30% (Sino-Swed Pharmaceutical Corp. Ltd., Beijing, China) was diluted to fifteen levels (0%, 1%, 2%, 3%, 4%, 5%, 6%, 7%, 8%, 9%, 10%, 11%, 12%, 13%, and 14%) with pure water in plastic vials to constitute a series of standard fat–water mixtures with known fat concentrations.

### 2.3. CSI

The standard fat–water mixtures and the pancreas were scanned using a 3.0 T magnetic resonance scanner (MAGNETOM Skyra, Siemens Healthcare, Erlangen, Germany) with the parameters previously reported [22,27]. A T1-weighted two-dimensional spoiled double-echo gradient-echo sequence was used to obtain in-phase (IP) and opposed-phase (OP) images. The repetition time (TR)/echo time (TE) for IP and OP images were 80/2.46 milliseconds and 80/1.23 milliseconds, respectively, with a flip angle of 50°. The whole pancreas was scanned with the following parameters: number of slices: 24; section thickness: 5.0 mm; intersection gap: 1.0 mm; matrix size: 352 × 286; and field of vision: 415 × 335 mm^2^. The scan time was 15 s within a single breath hold. A multi-echo spoiled gradient-echo sequence was used to estimate the equal T2* of fat and water with the following parameters: TR: 9.15 milliseconds; TEs: 2.46, 3.69, 4.92, 6.15, and 7.38 milliseconds; flip angle: 4°; section thickness: 5 mm; matrix size: 160 × 95; field of vision: 420 × 315 mm^2^; and scan time: 13 s within a single breath hold. All magnetic resonance scanning was accomplished by an experienced technician.

### 2.4. Image Analysis

All image analysis was completed by a radiologist who was blinded to the clinical data of the participants. To ensure the reliability and reproducibility of the quantitative measurements, the intra-observer reliability was assessed by randomly re-analyzing 20% of all MRI scans at least 2 weeks apart. The intraclass correlation coefficient (ICC) was calculated, and excellent intra-observer reliability was confirmed (ICC > 0.90). The ROI placement was performed per the standardized protocol detailed in the manuscript to minimize subjective bias. The ROI’s signal intensity (SI) was calculated for the IP and OP images. The ROIs for the standard fat–water mixture were set as 80 to 100 mm^2^. The ROIs for the pancreas were set as 40 to 60 mm^2^ to avoid pancreatic ducts, large blood vessels, and peripancreatic fat. For each vial of standard fat–water mixture, 9 ROIs were acquired from 3 different layers, with 3 ROIs in each layer. For each region of the pancreas (caput, corpus, and cauda), 9 ROIs were drawn from 3 different layers, with 3 ROIs in each layer. The mean fat fraction of the whole pancreas was determined using the averaged fat fractions of the caput, corpus, and cauda. Fat fractions with T1 and T2* relaxation corrections (*FF*_*T*1*T*2*corrected_) were determined according to Formulas (1)–(3):

(1) $$FF_{T1T2*correction}=\frac{a*(SI_{ip}*e^{y}-SI_{op}*e^{x})}{SI_{ip}*e^{y}*(a+b)+SI_{op}*e^{x}*(b-a)}$$

(2) $$a,b=\sin\alpha *\frac{1-e^{-TR/T1_{wf}}}{1-\cos\alpha *e^{-TR/T1_{wf}}}$$

(3) $$e^{x,y}=e^{-TE_{ip.op}/T2*}$$

where *SI_ip_* denotes the SI in IP images; *SI_op_* denotes the SI in OP images; and *w*, *f*, and *α* indicate the water, fat, and excitation flip angles, respectively.

CV is a dimensionless indicator that was used to represent the relative dispersion of fat fractions among the pancreatic caput, corpus, and cauda, allowing for an objective comparison of the heterogeneity between groups with different mean fat levels.

To quantify the spatial heterogeneity of the pancreatic fat distribution, we also calculated the absolute difference in fat fraction (ΔFF) between the regions with the highest and lowest fat fractions within the pancreas (ΔFF = FFmaximum − FFminimum).

### 2.5. Statistical Analysis

Before conducting parameter testing (one-way ANOVA), the Shapiro–Wilk test was used to evaluate the normality of the data. The results show that the pancreatic fat fraction and coefficient of variation (CV) followed normal distributions (*p* > 0.05), meeting the applicable conditions for parameter testing. Data were expressed as the mean ± standard deviation (SD) and were analyzed using SPSS 13.0 software (SPSS, Chicago, IL, USA). The association between the calculated and actual fat fractions was analyzed using Pearson’s correlation and linear regression analysis. One-way ANOVA was used to investigate the differences in pancreatic fat content between different groups. The coefficient of variation (CV) was analyzed using the *u* test. A two-sided *p*-value < 0.05 was considered statistically significant.

### 2.6. Machine Learning Analysis

To further explore the relative importance of potential predictors and their nonlinear associations with pancreatic steatosis (PS, %), a random forest regression model was constructed using Python 3.9 (scikit-learn 1.2.2, SHAP 0.41.0). The predictors included age (continuous variable) and drinking status (binary: 0 = non-drinker, 1 = drinker). The outcome variable was the whole pancreatic fat fraction (PS, %). The variable importance was used to quantify the contribution of each predictor, and SHAP values were computed to interpret the model predictions.

## 3. Results

### 3.1. Phantom Study

The calculated fat fractions (*FF_T_*_1*T*2*corrected_) of the 15 standard fat–water mixtures were linearly and positively correlated with the actual fat fractions (R^2^ = 0.991; *p* < 0.001). The relationship between the actual ($\hat{Y}$) and calculated fat fractions ($\hat{X}$) is given as a linear regression equation (*p* < 0.001): $\hat{Y}$ = 0.9679$\hat{X}\;$− 3.6064 (3.26 ≤ *X* ≤ 18.36). Using this linear regression equation, pancreatic fat fractions could be calculated from *FF_T_*_1*T*2*corrected_.

### 3.2. Effect of Long-Term Alcohol Intake on Distribution of Pancreatic Fat

**Figure 1:**
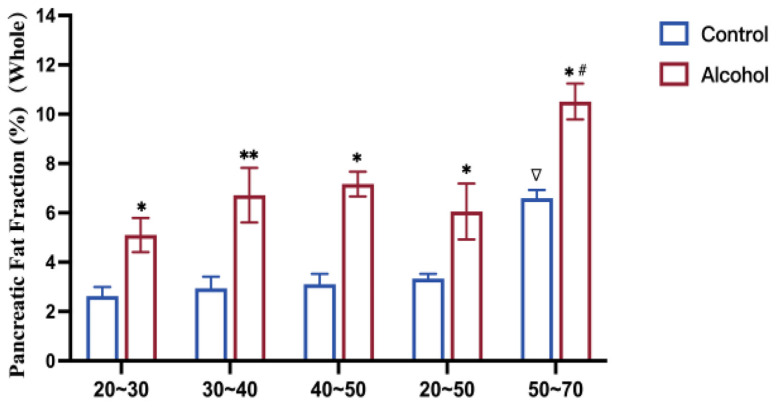
Pancreatic fat fraction in volunteers (20~70 years). Pancreatic fat fraction in different age groups and alcohol consumption states. Compared with the control group at different ages, ∇ *p* < 0.05. Compared with the alcohol group at different ages, # *p* < 0.05. Compared with the control group at the same age, * *p* < 0.05, ** *p* < 0.01.

**Figure 2:**
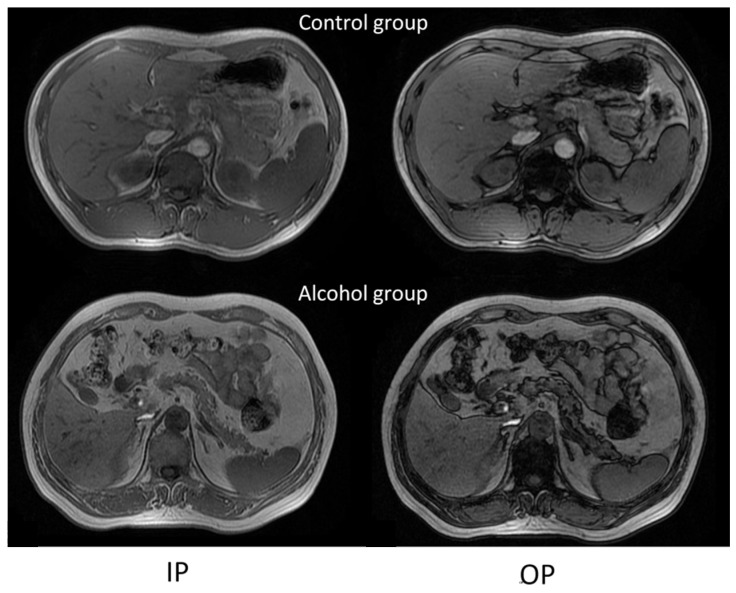
Images of the pancreas in volunteers aged 40–70 years (CSI). IP: in-phase; OP: opposed-phase; CSI: chemical shift magnetic resonance imaging; SI: signal intensity. The SI of the pancreas in the control group was even, but the SI of the alcoholic pancreas was heterogeneous. The pancreas of alcoholics demonstrated heterogeneous signal intensity with irregular, stippled margins on CSI.

The pancreatic fat fraction showed no significant difference between the caput, corpus, cauda, and whole pancreas in the control and alcohol groups at all ages (Table 1, Figure 1). Moreover, the fat fractions’ CV in the caput, corpus, cauda, and whole pancreas showed no obvious difference between the alcoholics and non-drinkers aged 20~30 and 30~40 years (*p* > 0.05, Table 2). However, the fat fractions’ CV in different pancreatic regions of alcoholics aged 40~50 and 50~70 years was significantly increased compared with those of the control group at the same age (*p* = 0.019, Table 2). Compared with normal controls, drinkers over the age of 40 have an uneven signal intensity within the pancreas and on the outer edge of the pancreas (Figure 2).

In addition to the coefficient of variation (CV), we also analyzed the absolute difference ΔFF between the highest and lowest fat fraction regions within the pancreas to quantify the spatial heterogeneity. For drinkers aged 40–50, the ΔFF was 2.34 ± 0.87% (range: 1.02–4.15%), which was significantly higher than that of the control group of the same age (1.12 ± 0.43%, range: 0.56–2.03%, *p* < 0.018). Similarly, the ΔFF for drinkers aged 50–70 (2.89 ± 1.05%, range: 1.21–4.98%) was significantly higher than that of the control group (1.35 ± 0.51%, range: 0.62–2.41%, *p* = 0.011).

### 3.3. Effect of Age on Pancreatic Fat Content

As listed in Table 1, the pancreatic fat fraction of volunteers aged 50~70 years was markedly higher than that of volunteers aged 20~50 years in the alcohol and control groups (9.14 ± 2.22 vs. 6.07 ± 1.59 and 5.98 ± 1.00 vs. 2.94 ± 0.62%, respectively; *p* = 0.016). However, there is no significant difference in pancreatic fat fraction between the 20~30-, 30~40-, and 40~50-year-old groups in the controls and alcoholics.

### 3.4. Effect of Chronic Ethanol Intake on Pancreatic Fat Content

The pancreatic fat fraction of alcoholics aged 20~50 years was about double that of the control group at the same age (6.07 ± 1.59% vs. 2.94 ± 0.62%, *p =* 0.026). Alcoholics aged 50~70 years also showed a significantly higher pancreatic fat fraction than the peer control group (9.14 ± 2.22% vs. 5.98 ± 1.00%, *p =* 0.021) (Table 1, Figure 2).

### 3.5. Effect of Abstinence on Pancreatic Fat Fraction

There was no significant difference in the pancreatic fat fraction between the current drinkers and abstainers (*p* > 0.05, Table 3). When abstainers were divided into short-term abstainers who stopped drinking for <1 year and long-term abstainers who stopped drinking for 1–2 years, there was still no statistical difference between the short-term abstainers, long-term abstainers, and current drinkers regarding pancreatic fat.

### 3.6. Machine Learning Analysis Results

**Figure 3:**
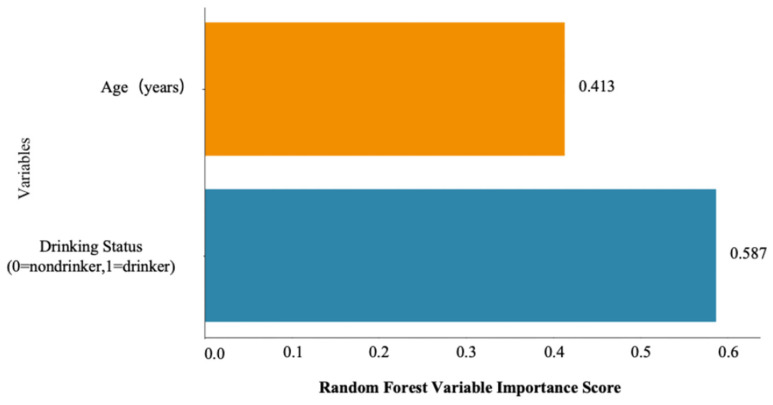
Random forest variable importance for pancreatic steatosis. The random forest variable importance plot for PS shows that drinking status was the dominant predictor (importance score = 0.587), followed by age (importance score = 0.413). These results are consistent with our main findings that chronic alcohol consumption is the major contributor to pancreatic steatosis (blue = drinking status, orange = age).

**Figure 4:**
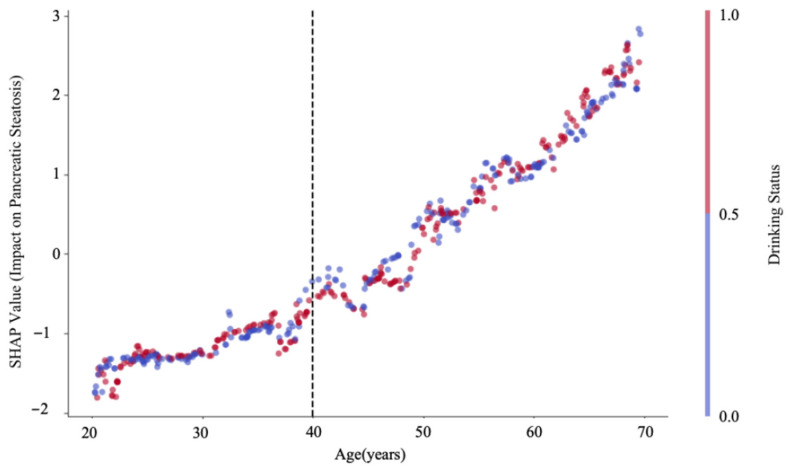
SHAP dependence plot: age vs. pancreatic steatosis (PS). SHAP dependence plot illustrating the association between age and pancreatic steatosis (PS). The plot demonstrates a consistent positive association between age and PS (SHAP values increased with age across 20–70 years). The vertical dashed line at 40 years indicates a slight inflection point, suggesting a weakly nonlinear relationship between age and PS. The color gradient from blue to red represents drinking status (blue = non-drinker, red = drinker).

Based on the variable importance analysis in Figure 3, drinking status was identified as the most important predictor (importance score = 0.587), followed by age (importance score = 0.413). In addition, the SHAP dependence plot verified a positive and weakly nonlinear association between age and pancreatic steatosis (PS) (Figure 4).

## 4. Discussion

In previous studies, alcohol-induced pancreatic steatosis was demonstrated in animals and human cadavers through histology and biochemical assays, which confirmed lipid droplet accumulation and increased triglyceride content [12,13,18]. However, the imaging features of alcoholic fatty pancreas in living people have rarely been reported. In this study, we identified alcohol-induced pancreatic steatosis in the male population using the CSI technique. The pancreatic fat fraction in alcoholics was about 1.5~2 times higher than that of age-matched non-drinkers.

To minimize confounding, female participants were not enrolled due to the potential influence of menopausal status on pancreatic fat deposition [22]. The non-drinkers and alcoholics were comparable in age and BMI, which are established risk factors for fatty pancreas [3,28,29,30,31]. Moreover, volunteers with pancreatic diseases, hyperlipidemia, diabetes mellitus, pre-diabetes, and metabolic syndrome were excluded, as these conditions have been associated with pancreatic fat accumulation [23,24,25,26,28,32,33,34,35]. The results suggest that chronic alcohol consumption is a major contributor to pancreatic fat deposition in the male population.

Consistent with our previous research that demonstrated age-related pancreatic fat accumulation after age 50 in healthy males [23], the present study observed a notable increase in pancreatic fat among volunteers aged 50–70 years. These findings indicate that alcoholics aged over 50 are likely to have fatty pancreas under the double effects of alcohol consumption and senescence. Moreover, alcohol consumption probably contributes to premature pancreatic aging in males aged 20~50 years, as their pancreatic fat fraction was comparable with that of non-alcoholics aged 50~70 years. Machine learning analysis consistently verified that drinking status was the strongest predictor of pancreatic steatosis, while age exhibited a positive, weakly nonlinear association. The interaction between drinking status and age further amplified the pancreatic fat accumulation.

Under normal conditions, fat is distributed uniformly in the pancreas [22,23,36,37]. In this study, both young and aged non-alcoholics exhibited homogeneous pancreatic fat distribution, suggesting that aging alone (>50 years) increased the pancreatic fat content but did not alter its fat distribution. In contrast, alcoholics aged 40~70 years demonstrated a heterogeneous fat deposition, as quantified by the coefficient of variation in pancreatic fat content. Using the CSI, the peripancreatic region in severe cases exhibited heterogeneous signal intensity with irregular and stippled margins.

The mechanisms underlying this uneven fat distribution and signal intensity on CSI still need further investigation and clarification through imaging, histology, or molecular analysis. Similar to alcohol-induced hepatic fat deposition, which is most severe in periportal areas due to initial and maximal alcohol exposure, we speculate that pancreatic injury may also be uneven and potentially associated with the regional arterial supply [38,39]. The heterogeneous fat deposition in drinkers may be related to the anatomical and metabolic characteristics of the pancreas. Unlike hepatocytes, which have high lipid transport and catabolic capabilities, pancreatic acinar cells lack lipid droplets under physiological conditions and have limited ability to decompose and accumulate triglycerides [40]. Additionally, the pancreas receives blood from multiple arteries (gastroduodenal, superior mesenteric artery, and splenic artery), potentially resulting in an uneven exposure of pancreatic parenchyma to ethanol metabolites [39]. This uneven exposure, combined with the inherent metabolic limitations of acinar cells, leads to focal fat accumulation. Furthermore, the downregulation of alcohol-induced pancreatic lipases (ATGL and HSL) further impairs lipid clearance, exacerbating the persistence of heterogeneous fat deposition [20]. These metabolic differences between pancreatic acinar cells and hepatocytes may explain why alcoholic pancreatic steatosis is more stubborn than alcoholic fatty liver disease. Furthermore, aging-related atrophy of pancreatic cells contributes to fat infiltration in the pancreas [41,42]. Heterogeneous fat deposition may lead to regional differences in pancreatic exocrine function, as focal fat accumulation can compress acinar cells and impair enzyme secretion [43]. This regional dysfunction may increase the risk of acute pancreatitis by promoting pancreatic duct obstruction or altering local inflammatory responses [18,44,45]. Future studies should evaluate the correlation between pancreatic exocrine function and fat distribution patterns. Although previous studies have identified the pancreatic body and tail as primary sites of fatty degeneration [23,46], this pattern was not observed in our cohort, highlighting the need for further research to confirm the mechanisms underlying alcohol-induced uneven pancreatic fat deposition.

While abstinence effectively attenuates alcoholic fatty liver in animal models and humans [47], alcohol-induced pancreatic fat deposition in rats had not reversed after 3 months of alcohol withdrawal [12]. Consistent with these results in animals, our human study showed that the pancreatic fat content of abstainers who refrained from alcohol for <1 year or 1~2 years was still significantly higher than that of controls and remained at the level of current drinkers in the present human study. This suggests alcoholic fatty pancreas is more stubborn than alcoholic fatty liver, which typically shows marked improvement after 4–6 weeks of abstinence. Currently, no effective treatment for alcoholic fatty pancreas has been reported. Within the observed abstinence period of up to two years, we found no significant reversal of alcohol-induced pancreatic fat deposition. Our previous animal study revealed that this irreversibility may be attributed to reduced pancreatic cholecystokinin (CCK), long-standing fatty infiltration, and ultrastructural injuries in pancreatic acinar cells [12]. We also found that betaine can restore the rat pancreatic adiponectin signaling pathway, directly protecting acinar cells from ethanol-induced pancreatic overactivation, reducing the increase in pancreatic TG and FFA levels caused by alcohol consumption, as well as mitigating damage to lipid droplets and organelle microstructures [48]. However, whether betaine can reverse existing fat deposition in the human pancreas still requires clinical trial validation. Therefore, preventing first-time alcohol consumption and developing effective therapeutic strategies remain critical priorities.

There are several limitations to the current study. First, this study adopted a cross-sectional design, which only demonstrated the association between chronic alcohol consumption and pancreatic steatosis, rather than a definite causal relationship. Therefore, it is impossible to draw conclusions about the causal direction of this relationship from the present findings. Second, this was not a multicenter study. Third, female volunteers were not recruited, which may be related to the potential influence of menopause on pancreatic fat scores. Fourth, there was no histological confirmation of pancreatic fat fraction and distribution. Fifth, alcohol consumption was evaluated solely through self-reporting, without biochemical validation using objective biomarkers such as carbohydrate-deficient transferrin (CDT) and phosphatidylethanol (PEth). Sixth, the longest abstinence period of 2 years was not sufficient to support a clear conclusion about the irreversibility of pancreatic steatosis, and there is no literature on the effects of quitting drinking for more than two years on alcoholic fatty pancreas that can currently alleviate this limitation. Therefore, future research can include both male and female populations to obtain more comprehensive conclusions, validate alcohol exposure levels by integrating objective biomarkers, optimize research plans, and reduce potential biases caused by self-reporting. At the same time, it is necessary to extend the follow-up period for alcohol withdrawal (e.g., >5 years) to further clarify whether pancreatic steatosis is reversible. Mechanistic studies combined with histological validation will be performed to better elucidate the underlying pathological mechanisms. In addition, it is necessary to conduct clinical trials to evaluate the intervention effect of potential treatment methods, such as betaine, on alcoholic pancreatic steatosis.

## 5. Conclusions

In conclusion, chronic alcohol consumption could cause fat deposition in the pancreas. An uneven distribution of pancreatic fat began in the fourth decade in this male population. Alcoholic fatty pancreas was not reversed within a follow-up period of up to two years. These findings underscore that primary prevention through avoidance of alcohol consumption remains the most effective current strategy against alcoholic pancreatic steatosis.

## Figures and Tables

**Table 1:** Pancreatic fat fraction in volunteers (20~70 years).

Age	Group Pancreatic Fat Fraction (%)				
(Years)	(*n*)	Caput	Corpus	Cauda	Whole
20~30	Control (32)	2.55 ± 0.42	2.91 ± 0.66	2.35 ± 0.62	2.61 ± 0.60
	Alcohol (36)	4.95 ± 1.10 *	5.42 ± 1.50 *	4.51 ± 0.96 *	5.00 ± 1.07 *
30~40	Control (35)	2.54 ± 0.60	3.08 ± 0.77	3.11 ± 0.84	2.91 ± 0.66
	Alcohol (31)	6.31 ± 1.42 **	7.38 ± 1.98 **	6.00 ± 1.67 **	6.55 ± 1.57 **
40~50	Control (37)	3.18 ± 0.79	2.81 ± 0.67	3.13 ± 0.63	3.09 ± 0.57
	Alcohol (39)	6.20 ± 2.25 *	6.30 ± 2.15 *	5.90 ± 1.71 *	6.14 ± 1.61 *
20~50	Control (104)	3.02 ± 0.95	3.03 ± 0.81	2.77 ± 0.84	2.94 ± 0.62
	Alcohol (106)	5.96 ± 1.89 *	6.59 ± 1.80 **	5.63 ± 1.62 *	6.07 ± 1.59 *
50~70	Control (38)	5.97 ± 1.54 ^∇^	6.36 ± 1.31 ^∇^	5.75 ± 1.26 ^∇^	5.98 ± 1.00 ^∇^
	Alcohol (34)	9.34 ± 3.54 *^#^	9.26 ± 2.77 *^#^	8.88 ± 2.88 *^#^	9.14 ± 2.22 *^#^

The fat fraction of the whole pancreas was calculated from the average of the fat fractions in the caput, corpus, and cauda of the pancreas. Compared with the control group at different age ranges (20~50 years vs. 50~70 years), the 50–70 years subgroup showed significant differences (*p* < 0.05): caput (*p* = 0.034), corpus (*p* = 0.023), cauda (*p* = 0.029), whole pancreas (*p* = 0.027). Compared with the alcohol group aged 20~50 years (vs. 50~70 years), the 50~70 years subgroup showed significant differences (# *p* < 0.05): caput (*p* = 0.018), corpus (*p* = 0.011), cauda (*p* = 0.029), whole pancreas (*p* = 0.033). Compared with the control group at the same age (* *p* < 0.05, ** *p* < 0.01): 20~30 y: caput (*p* = 0.012), corpus (*p* = 0.022), cauda (*p* = 0.018), whole (*p* = 0.020); 30~40 y: caput (*p* = 0.002), corpus (*p* = 0.004), cauda (*p* = 0.006), whole (*p* = 0.001); 40~50 y: caput (*p* = 0.021), corpus (*p* = 0.014), cauda (*p* = 0.026), whole (*p* = 0.020); 20~50 y: caput (*p* = 0.018), corpus (*p* = 0.003), cauda (*p* = 0.019), whole (*p* = 0.027); 50~70 y: caput (*p* = 0.012), corpus (*p* = 0.025), cauda (*p* = 0.011), whole (*p* = 0.019).Compared with the control group at different age ranges (20–50 years vs. 50–70 years), the 50–70 years subgroup showed significant differences (∇ *p* < 0.05): caput (*p* = 0.034), corpus (*p* = 0.023), cauda (*p* = 0.029), whole pancreas (*p* = 0.027).

**Table 2:** Coefficient of variation (CV) of pancreatic fat fractions.

	CV				
Age (Years)	Group (*n*)	Caput	Corpus	Cauda	Whole
20~30	Control (32)	0.164	0.227	0.262	0.230
	Alcohol (36)	0.223	0.278	0.213	0.214
30~40	Control (35)	0.236	0.249	0.269	0.226
	Alcohol (31)	0.225	0.268	0.279	0.240
40~50	Control (37)	0.249	0.238	0.201	0.185
	Alcohol (39)	0.362 *	0.342 *	0.289 *	0.262 *
50~70	Control (38)	0.258	0.206	0.219	0.167
	Alcohol (34)	0.379 *	0.299 *	0.324 *	0.243 *

Compared with the control group at the same age (* *p* < 0.05), 40~50 y: caput (*p* = 0.021), corpus (*p* = 0.019), cauda (*p* = 0.024), whole (*p* = 0.022); 50~70 y: caput (*p* = 0.034), corpus (*p* = 0.013), cauda (*p* = 0.027), whole (*p* = 0.015).

**Table 3:** Effect of abstinence on pancreatic fat fraction.

Age (Years) (*n*)	Fat Fraction in the Whole Pancreas (%)			
	Current Drinkers (79)	All Abstainers (61)	Abstainers <1 Year (31)	Abstainers 1–2 Years (30)
20~50 (106)	6.38 ± 1.33	5.71 ± 1.61	5.93 ± 1.47	5.44 ± 1.29
50~70 (34)	8.52 ± 1.95	9.92 ± 2.65	9.64 ± 2.46	10.48 ± 2.87

## Data Availability

The data are available from the corresponding author upon reasonable request.

## Supplementary Materials

The following supporting information can be downloaded at: https://www.mdpi.com/article/10.3390/jcm15072513/s1, Table S1: Inclusion and exclusion criteria for enrollment.
