# Supplementary material for: Chronic Alcohol Consumption Induces Irreversible and Heterogeneous Pancreatic Steatosis in Men: An MRI-Based Cross-Sectional Study
Source: J Clin Med. 2026 Mar 25;15(7):2513. doi: 10.3390/jcm15072513 (PMC13073420; doi:10.3390/jcm15072513)
Supplement: Supplementary file 1 [file jcm-15-02513-s001.zip › jcm-4138223-supplementary.pdf]

TABLES

Table S1. Inclusion and exclusion criteria for enrollment.

**Inclusion Criteria**
Age: 20~70 years old
Gender: Male
BMI: 18.5~24.9 kg/m^2
Drinking history: non-drinkers (the people without history of long-term or excess alcohol consumption (>20 g ethanol/day for at least one year)) or alcoholics (the people who drink for more than 5 years with consumption of at least 20 g ethanol in one day and do not stop drinking for more than 2 years)
**Exclusion Criteria**
Severe cardiac or pulmonary diseases, renal dysfunction, autoimmune diseases, malignant tumors, severe infection;
Hyperlipemia, diabetes mellitus, pre-diabetes, metabolic syndrome;
Acute pancreatitis, chronic pancreatitis, pancreatic cystic disease, pancreatic cancer, gallstones, biliary infection, cholecystitis, cholangitis, sphincter of Oddi disease, cirrhosis, hepatitis, hepatic metabolic disorders, hepatic vascular diseases, portal hypertension, post-liver transplantation, peptic ulcer, inflammatory bowel disease, bowel obstruction, ischemic lesions of the bowel, or surgeries of the digestive system;
History or current use of glucocorticosteroids, insulin, and/or thiazolidinediones;;
Pancreatic enzyme or proton pump inhibitor therapy in the 3 months prior to enrollment
Weight change in nearly three months ≥ 5%

Body mass index (BMI) is calculated by dividing weight in kilograms by height in meters squared.
